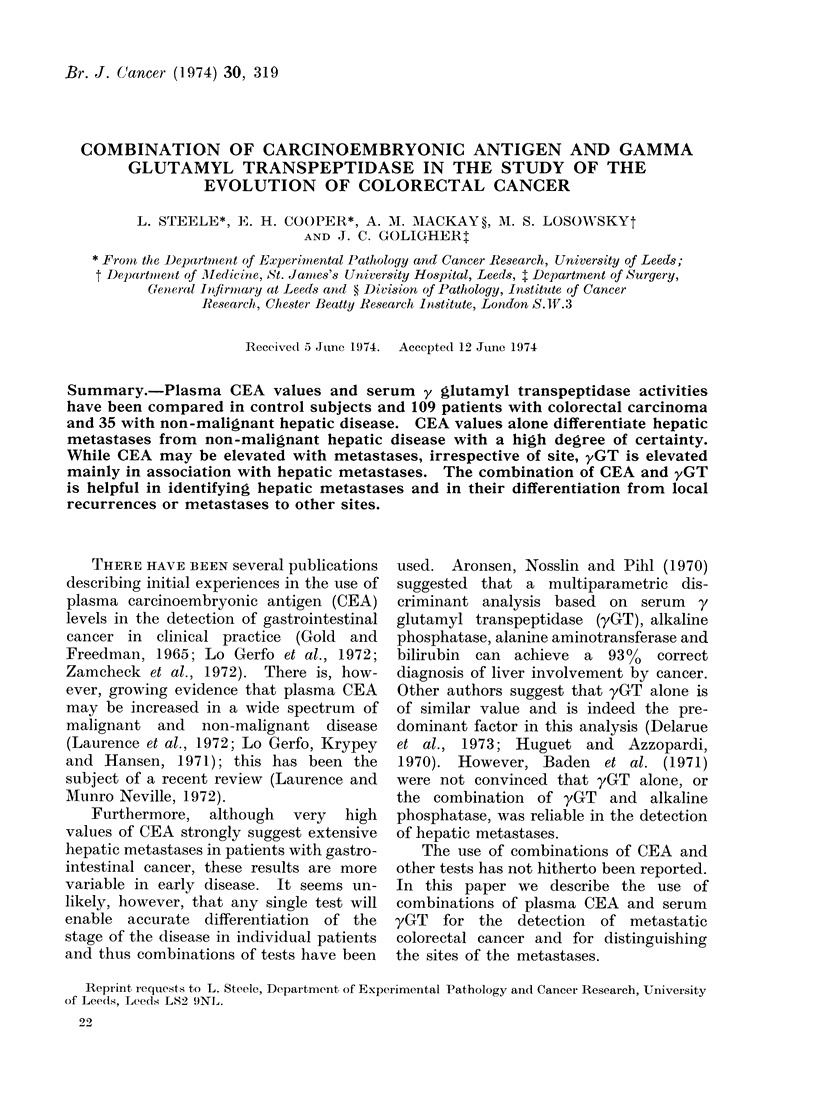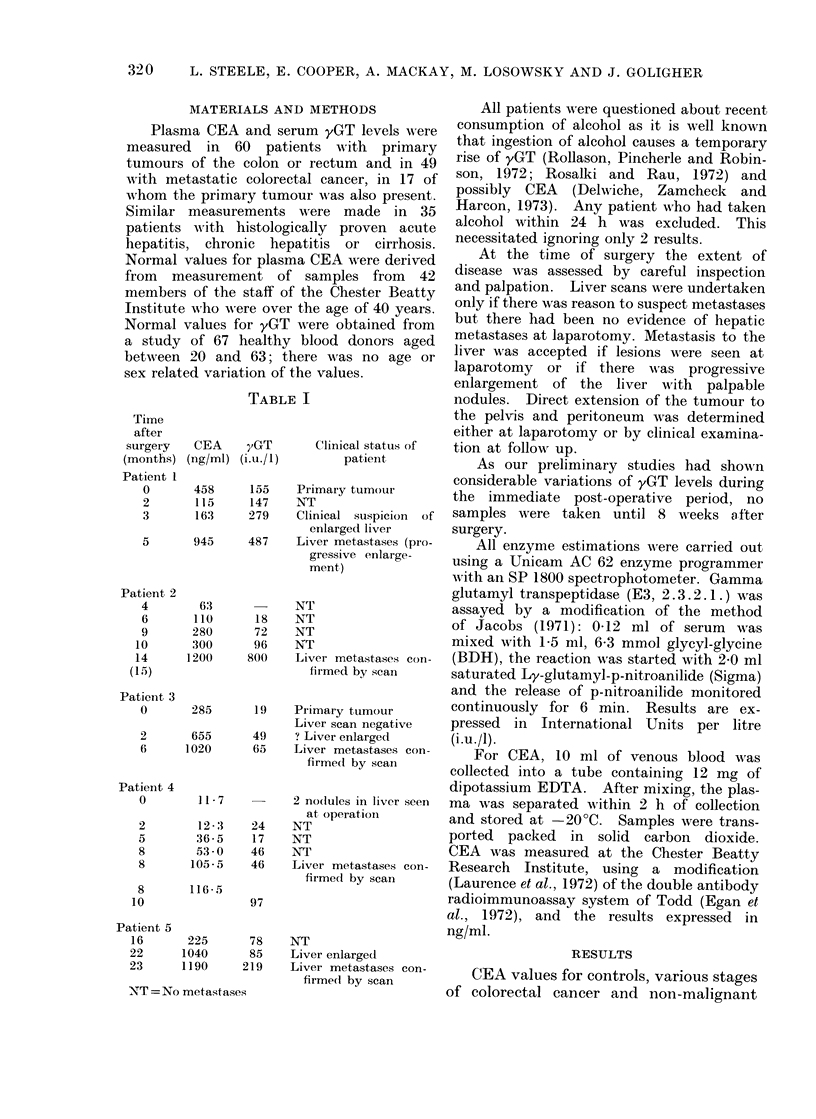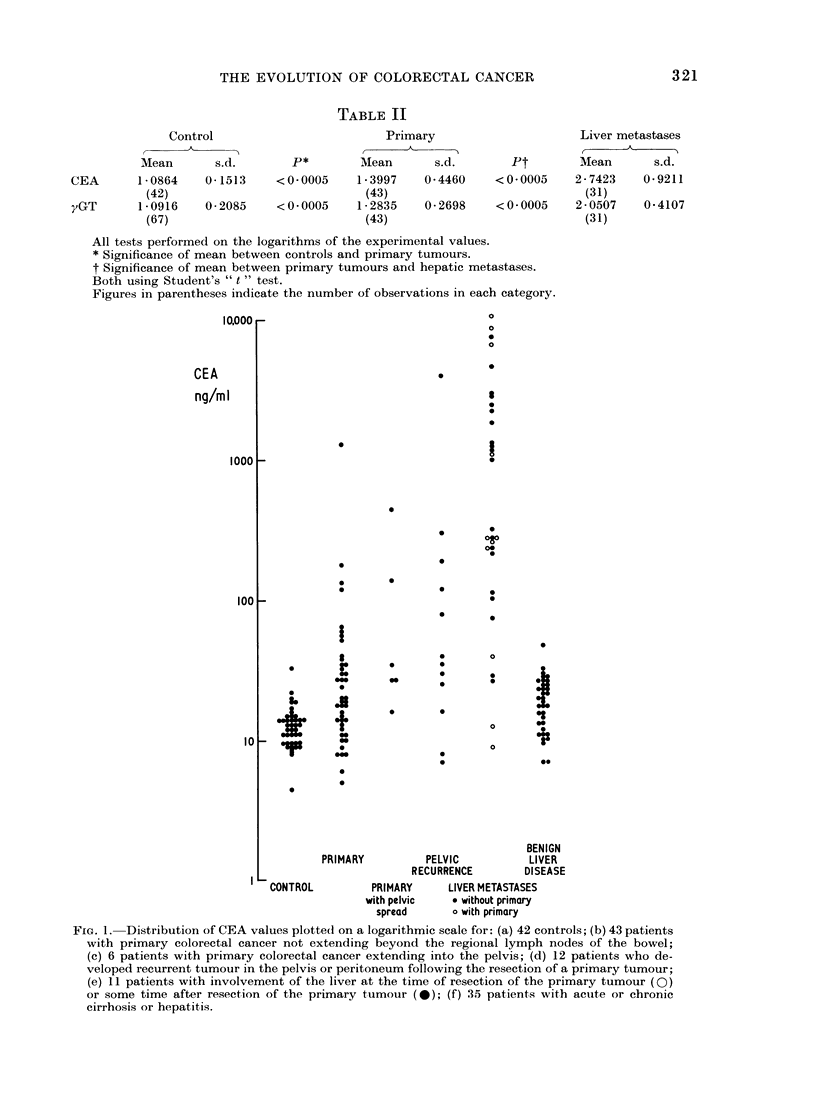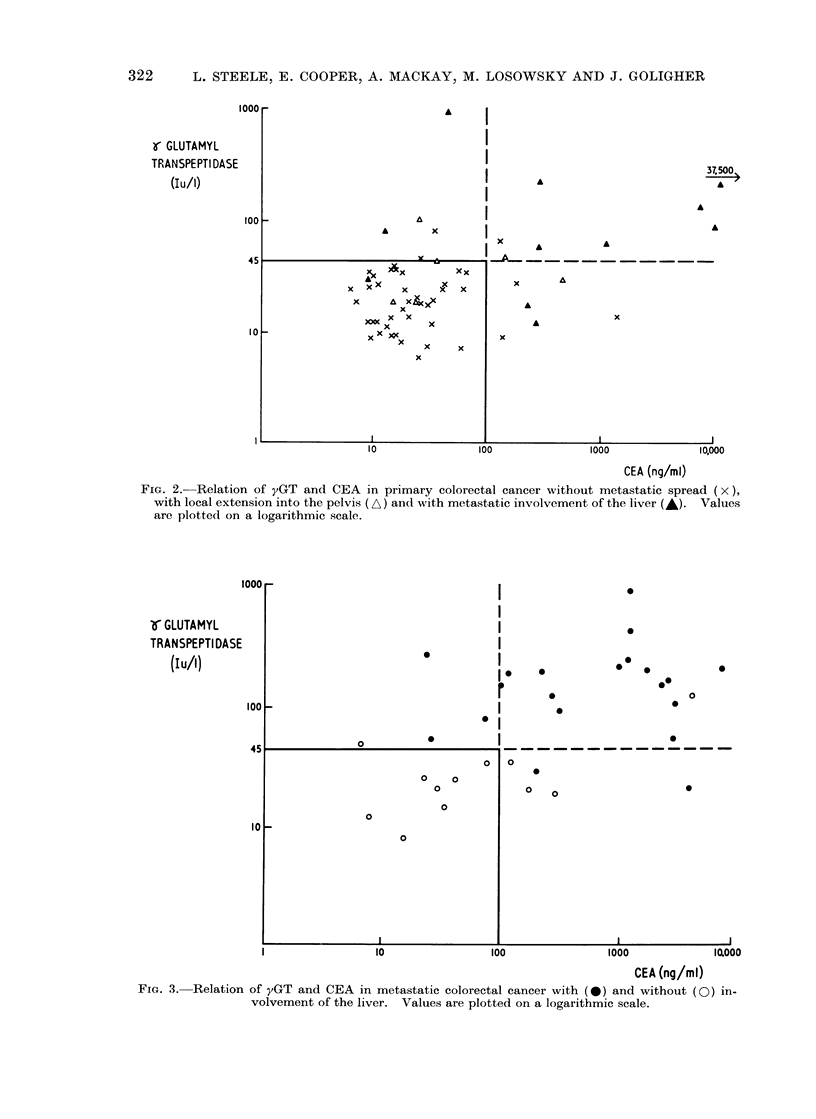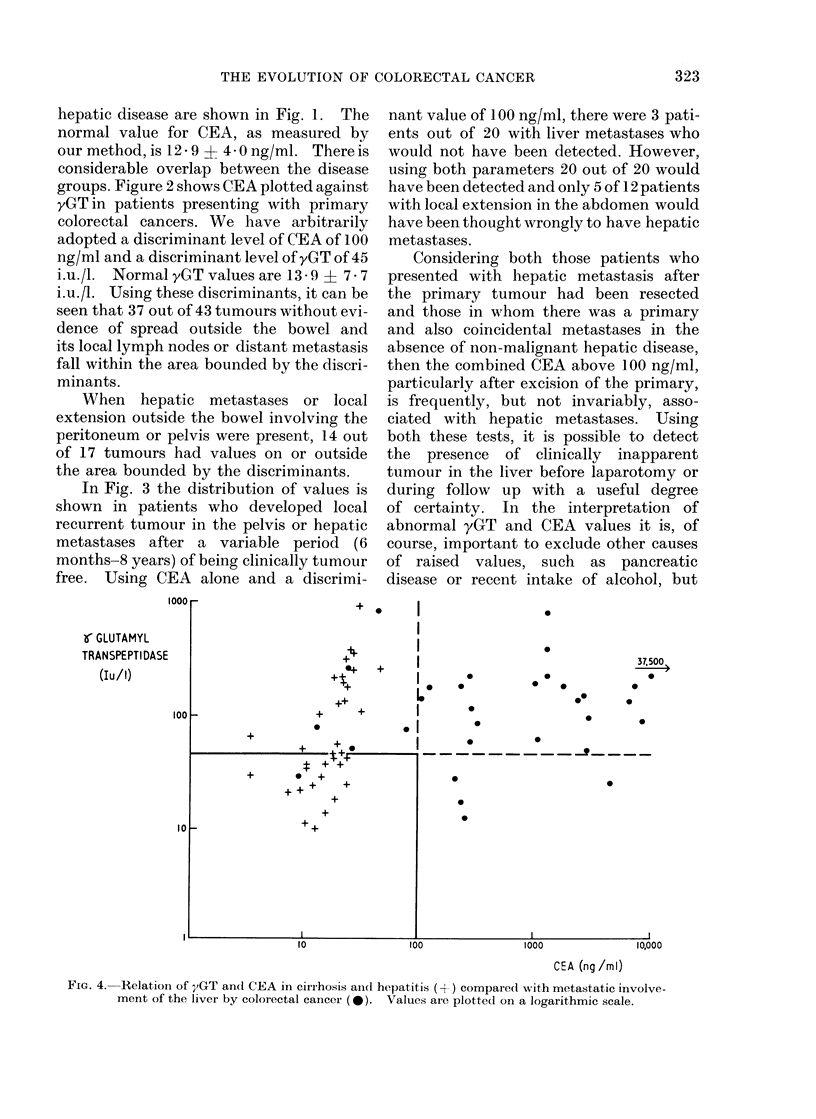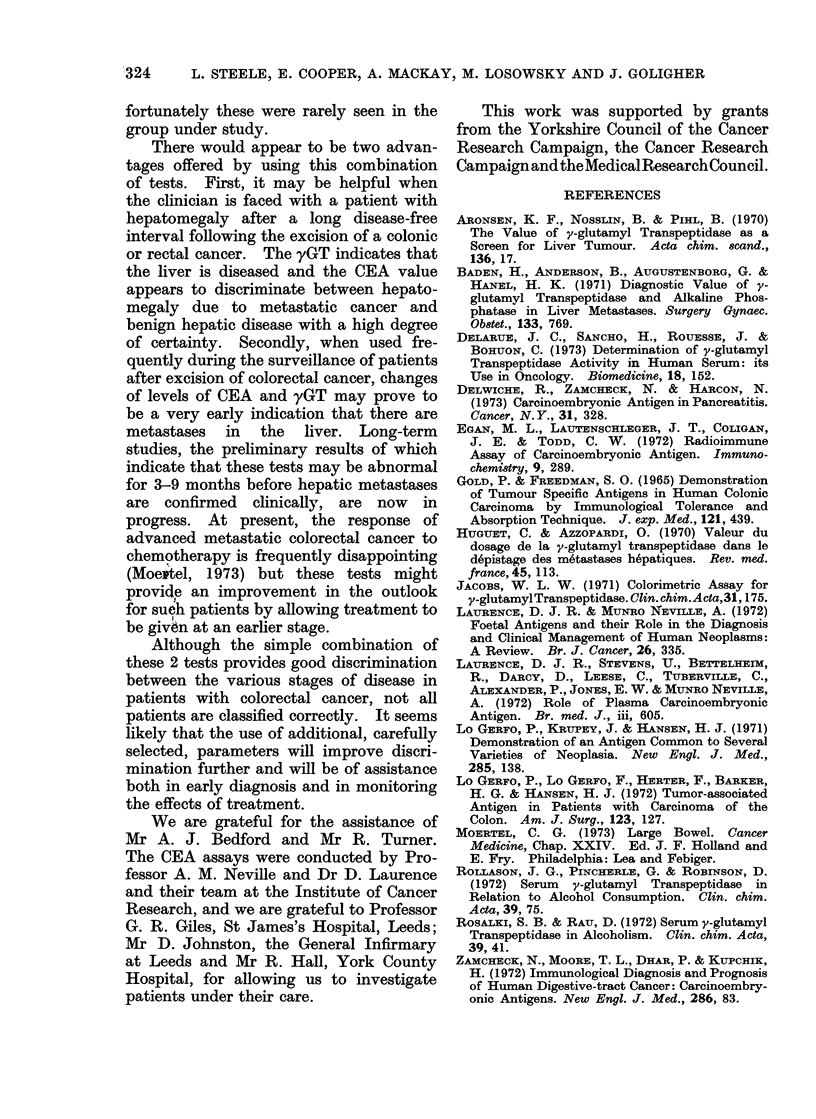# Combination of Carcinoembryonic Antigen and Gamma Glutamyl Transpeptidase in the Study of The Evolution of Colorectal Cancer

**DOI:** 10.1038/bjc.1974.199

**Published:** 1974-10

**Authors:** L. Steele, E. H. Cooper, A. M. Mackay, M. S. Losowsky, J. C. Goligher

## Abstract

Plasma CEA values and serum γ glutamyl transpeptidase activities have been compared in control subjects and 109 patients with colorectal carcinoma and 35 with non-malignant hepatic disease. CEA values alone differentiate hepatic metastases from non-malignant hepatic disease with a high degree of certainty. While CEA may be elevated with metastases, irrespective of site, γGT is elevated mainly in association with hepatic metastases. The combination of CEA and γGT is helpful in identifying hepatic metastases and in their differentiation from local recurrences or metastases to other sites.


*Br. J. Cancer* (1974) **30**, 319

### COMBINATION OF CARCINOEMBRYONIC ANTIGEN AND GAMMA GLUTAMYL TRANSPEPTIDASE IN THE STUDY OF THE EVOLUTION OF COLORECTAL CANCER

L. STEELE*, E. H. COOPER*, A. M. MACKAY§, M. S. LOSOWSKY† AND J. C. GOLIGHER‡

** From the Department of Experimental Pathology and Cancer Research, University of Leeds; † Department of Medicine, St. James's University Hospital, Leeds, ‡ Department of Surgery, General Infirmary at Leeds and § Division of Pathology, Institute of Cancer Research, Chester Beatty Research Institute, London S.W.3*

Received 5 June 1974. Accepted 12 June 1974

**Summary.—Plasma CEA values and serum $\gamma$ glutamyl transpeptidase activities have been compared in control subjects and 109 patients with colorectal carcinoma and 35 with non-malignant hepatic disease. CEA values alone differentiate hepatic metastases from non-malignant hepatic disease with a high degree of certainty. While CEA may be elevated with metastases, irrespective of site, $\gamma$GT is elevated mainly in association with hepatic metastases. The combination of CEA and $\gamma$GT is helpful in identifying hepatic metastases and in their differentiation from local recurrences or metastases to other sites.**

THERE HAVE BEEN several publications describing initial experiences in the use of plasma carcinoembryonic antigen (CEA) levels in the detection of gastrointestinal cancer in clinical practice (Gold and Freedman, 1965; Lo Gerfo *et al.*, 1972; Zamcheck *et al.*, 1972). There is, however, growing evidence that plasma CEA may be increased in a wide spectrum of malignant and non-malignant disease (Laurence *et al.*, 1972; Lo Gerfo, Krypey and Hansen, 1971); this has been the subject of a recent review (Laurence and Munro Neville, 1972).

Furthermore, although very high values of CEA strongly suggest extensive hepatic metastases in patients with gastrointestinal cancer, these results are more variable in early disease. It seems unlikely, however, that any single test will enable accurate differentiation of the stage of the disease in individual patients and thus combinations of tests have been used. Aronsen, Nosslin and Pihl (1970) suggested that a multiparametric discriminant analysis based on serum $\gamma$ glutamyl transpeptidase ($\gamma$GT), alkaline phosphatase, alanine aminotransferase and bilirubin can achieve a 93% correct diagnosis of liver involvement by cancer. Other authors suggest that $\gamma$GT alone is of similar value and is indeed the predominant factor in this analysis (Delarue *et al.*, 1973; Huguet and Azzopardi, 1970). However, Baden *et al.* (1971) were not convinced that $\gamma$GT alone, or the combination of $\gamma$GT and alkaline phosphatase, was reliable in the detection of hepatic metastases.

The use of combinations of CEA and other tests has not hitherto been reported. In this paper we describe the use of combinations of plasma CEA and serum $\gamma$GT for the detection of metastatic colorectal cancer and for distinguishing the sites of the metastases.

Reprint requests to L. Steele, Department of Experimental Pathology and Cancer Research, University of Leeds, Leeds LS2 9NL.

### MATERIALS AND METHODS

Plasma CEA and serum γGT levels were measured in 60 patients with primary tumours of the colon or rectum and in 49 with metastatic colorectal cancer, in 17 of whom the primary tumour was also present. Similar measurements were made in 35 patients with histologically proven acute hepatitis, chronic hepatitis or cirrhosis. Normal values for plasma CEA were derived from measurement of samples from 42 members of the staff of the Chester Beatty Institute who were over the age of 40 years. Normal values for γGT were obtained from a study of 67 healthy blood donors aged between 20 and 63; there was no age or sex related variation of the values.

TABLE I

| Time after surgery (months) | CEA (ng/ml) | γGT (i.u./l) | Clinical status of patient |
|---|---|---|---|
| Patient 1 | | | |
| 0 | 458 | 155 | Primary tumour |
| 2 | 115 | 147 | NT |
| 3 | 163 | 279 | Clinical suspicion of enlarged liver |
| 5 | 945 | 487 | Liver metastases (progressive enlargement) |
| Patient 2 | | | |
| 4 | 63 | — | NT |
| 6 | 110 | 18 | NT |
| 9 | 280 | 72 | NT |
| 10 | 300 | 96 | NT |
| 14 (15) | 1200 | 800 | Liver metastases confirmed by scan |
| Patient 3 | | | |
| 0 | 285 | 19 | Primary tumour Liver scan negative |
| 2 | 655 | 49 | ? Liver enlarged |
| 6 | 1020 | 65 | Liver metastases confirmed by scan |
| Patient 4 | | | |
| 0 | 11·7 | — | 2 nodules in liver seen at operation |
| 2 | 12·3 | 24 | NT |
| 5 | 36·5 | 17 | NT |
| 8 | 53·0 | 46 | NT |
| 8 | 105·5 | 46 | Liver metastases confirmed by scan |
| 8 | 116·5 | | |
| 10 | | 97 | |
| Patient 5 | | | |
| 16 | 225 | 78 | NT |
| 22 | 1040 | 85 | Liver enlarged |
| 23 | 1190 | 219 | Liver metastases confirmed by scan |

NT = No metastases

All patients were questioned about recent consumption of alcohol as it is well known that ingestion of alcohol causes a temporary rise of γGT (Rollason, Pincherle and Robinson, 1972; Rosalki and Rau, 1972) and possibly CEA (Delwiche, Zamcheck and Harcon, 1973). Any patient who had taken alcohol within 24 h was excluded. This necessitated ignoring only 2 results.

At the time of surgery the extent of disease was assessed by careful inspection and palpation. Liver scans were undertaken only if there was reason to suspect metastases but there had been no evidence of hepatic metastases at laparotomy. Metastasis to the liver was accepted if lesions were seen at laparotomy or if there was progressive enlargement of the liver with palpable nodules. Direct extension of the tumour to the pelvis and peritoneum was determined either at laparotomy or by clinical examination at follow up.

As our preliminary studies had shown considerable variations of γGT levels during the immediate post-operative period, no samples were taken until 8 weeks after surgery.

All enzyme estimations were carried out using a Unicam AC 62 enzyme programmer with an SP 1800 spectrophotometer. Gamma glutamyl transpeptidase (E3, 2.3.2.1.) was assayed by a modification of the method of Jacobs (1971): 0·12 ml of serum was mixed with 1·5 ml, 6·3 mmol glycyl-glycine (BDH), the reaction was started with 2·0 ml saturated Lγ-glutamyl-p-nitroanilide (Sigma) and the release of p-nitroanilide monitored continuously for 6 min. Results are expressed in International Units per litre (i.u./l).

For CEA, 10 ml of venous blood was collected into a tube containing 12 mg of dipotassium EDTA. After mixing, the plasma was separated within 2 h of collection and stored at −20°C. Samples were transported packed in solid carbon dioxide. CEA was measured at the Chester Beatty Research Institute, using a modification (Laurence *et al.*, 1972) of the double antibody radioimmunoassay system of Todd (Egan *et al.*, 1972), and the results expressed in ng/ml.

### RESULTS

CEA values for controls, various stages of colorectal cancer and non-malignant

TABLE II

| | Control | | | Primary | | | Liver metastases | |
|---|---|---|---|---|---|---|---|---|
| | Mean | s.d. | $P$* | Mean | s.d. | $P$† | Mean | s.d. |
| CEA | 1·0864 (42) | 0·1513 | <0·0005 | 1·3997 (43) | 0·4460 | <0·0005 | 2·7423 (31) | 0·9211 |
| $\gamma$GT | 1·0916 (67) | 0·2085 | <0·0005 | 1·2835 (43) | 0·2698 | <0·0005 | 2·0507 (31) | 0·4107 |

All tests performed on the logarithms of the experimental values.
* Significance of mean between controls and primary tumours.
† Significance of mean between primary tumours and hepatic metastases.
Both using Student's "$t$" test.
Figures in parentheses indicate the number of observations in each category.

FIG. 1.—Distribution of CEA values plotted on a logarithmic scale for: (a) 42 controls; (b) 43 patients with primary colorectal cancer not extending beyond the regional lymph nodes of the bowel; (c) 6 patients with primary colorectal cancer extending into the pelvis; (d) 12 patients who developed recurrent tumour in the pelvis or peritoneum following the resection of a primary tumour; (e) 11 patients with involvement of the liver at the time of resection of the primary tumour (○) or some time after resection of the primary tumour (●); (f) 35 patients with acute or chronic cirrhosis or hepatitis.

FIG. 2.—Relation of γGT and CEA in primary colorectal cancer without metastatic spread (×), with local extension into the pelvis (△) and with metastatic involvement of the liver (▲). Values are plotted on a logarithmic scale.

FIG. 3.—Relation of γGT and CEA in metastatic colorectal cancer with (●) and without (○) involvement of the liver. Values are plotted on a logarithmic scale.

hepatic disease are shown in Fig. 1. The normal value for CEA, as measured by our method, is 12·9 ± 4·0 ng/ml. There is considerable overlap between the disease groups. Figure 2 shows CEA plotted against γGT in patients presenting with primary colorectal cancers. We have arbitrarily adopted a discriminant level of CEA of 100 ng/ml and a discriminant level of γGT of 45 i.u./l. Normal γGT values are 13·9 ± 7·7 i.u./l. Using these discriminants, it can be seen that 37 out of 43 tumours without evidence of spread outside the bowel and its local lymph nodes or distant metastasis fall within the area bounded by the discriminants.

When hepatic metastases or local extension outside the bowel involving the peritoneum or pelvis were present, 14 out of 17 tumours had values on or outside the area bounded by the discriminants.

In Fig. 3 the distribution of values is shown in patients who developed local recurrent tumour in the pelvis or hepatic metastases after a variable period (6 months–8 years) of being clinically tumour free. Using CEA alone and a discriminant value of 100 ng/ml, there were 3 patients out of 20 with liver metastases who would not have been detected. However, using both parameters 20 out of 20 would have been detected and only 5 of 12 patients with local extension in the abdomen would have been thought wrongly to have hepatic metastases.

Considering both those patients who presented with hepatic metastasis after the primary tumour had been resected and those in whom there was a primary and also coincidental metastases in the absence of non-malignant hepatic disease, then the combined CEA above 100 ng/ml, particularly after excision of the primary, is frequently, but not invariably, associated with hepatic metastases. Using both these tests, it is possible to detect the presence of clinically inapparent tumour in the liver before laparotomy or during follow up with a useful degree of certainty. In the interpretation of abnormal γGT and CEA values it is, of course, important to exclude other causes of raised values, such as pancreatic disease or recent intake of alcohol, but

FIG. 4.—Relation of γGT and CEA in cirrhosis and hepatitis (+) compared with metastatic involvement of the liver by colorectal cancer (●). Values are plotted on a logarithmic scale.

fortunately these were rarely seen in the group under study.

There would appear to be two advantages offered by using this combination of tests. First, it may be helpful when the clinician is faced with a patient with hepatomegaly after a long disease-free interval following the excision of a colonic or rectal cancer. The $\gamma$GT indicates that the liver is diseased and the CEA value appears to discriminate between hepatomegaly due to metastatic cancer and benign hepatic disease with a high degree of certainty. Secondly, when used frequently during the surveillance of patients after excision of colorectal cancer, changes of levels of CEA and $\gamma$GT may prove to be a very early indication that there are metastases in the liver. Long-term studies, the preliminary results of which indicate that these tests may be abnormal for 3–9 months before hepatic metastases are confirmed clinically, are now in progress. At present, the response of advanced metastatic colorectal cancer to chemotherapy is frequently disappointing (Moertel, 1973) but these tests might provide an improvement in the outlook for such patients by allowing treatment to be given at an earlier stage.

Although the simple combination of these 2 tests provides good discrimination between the various stages of disease in patients with colorectal cancer, not all patients are classified correctly. It seems likely that the use of additional, carefully selected, parameters will improve discrimination further and will be of assistance both in early diagnosis and in monitoring the effects of treatment.

We are grateful for the assistance of Mr A. J. Bedford and Mr R. Turner. The CEA assays were conducted by Professor A. M. Neville and Dr D. Laurence and their team at the Institute of Cancer Research, and we are grateful to Professor G. R. Giles, St James's Hospital, Leeds; Mr D. Johnston, the General Infirmary at Leeds and Mr R. Hall, York County Hospital, for allowing us to investigate patients under their care.

This work was supported by grants from the Yorkshire Council of the Cancer Research Campaign, the Cancer Research Campaign and the Medical Research Council.

### REFERENCES

ARONSEN, K. F., NOSSLIN, B. & PIHL, B. (1970) The Value of $\gamma$-glutamyl Transpeptidase as a Screen for Liver Tumour. *Acta chim. scand.*, **136**, 17.

BADEN, H., ANDERSON, B., AUGUSTENBORG, G. & HANEL, H. K. (1971) Diagnostic Value of $\gamma$-glutamyl Transpeptidase and Alkaline Phosphatase in Liver Metastases. *Surgery Gynaec. Obstet.*, **133**, 769.

DELARUE, J. C., SANCHO, H., ROUESSE, J. & BOHUON, C. (1973) Determination of $\gamma$-glutamyl Transpeptidase Activity in Human Serum: its Use in Oncology. *Biomedicine*, **18**, 152.

DELWICHE, R., ZAMCHECK, N. & HARCON, N. (1973) Carcinoembryonic Antigen in Pancreatitis. *Cancer, N.Y.*, **31**, 328.

EGAN, M. L., LAUTENSCHLEGER, J. T., COLIGAN, J. E. & TODD, C. W. (1972) Radioimmune Assay of Carcinoembryonic Antigen. *Immunochemistry*, **9**, 289.

GOLD, P. & FREEDMAN, S. O. (1965) Demonstration of Tumour Specific Antigens in Human Colonic Carcinoma by Immunological Tolerance and Absorption Technique. *J. exp. Med.*, **121**, 439.

HUGUET, C. & AZZOPARDI, O. (1970) Valeur du dosage de la $\gamma$-glutamyl transpeptidase dans le dépistage des métastases hépatiques. *Rev. med. france*, **45**, 113.

JACOBS, W. L. W. (1971) Colorimetric Assay for $\gamma$-glutamyl Transpeptidase. *Clin. chim. Acta*, **31**, 175.

LAURENCE, D. J. R. & MUNRO NEVILLE, A. (1972) Foetal Antigens and their Role in the Diagnosis and Clinical Management of Human Neoplasms: A Review. *Br. J. Cancer*, **26**, 335.

LAURENCE, D. J. R., STEVENS, U., BETTELHEIM, R., DARCY, D., LEESE, C., TUBERVILLE, C., ALEXANDER, P., JONES, E. W. & MUNRO NEVILLE, A. (1972) Role of Plasma Carcinoembryonic Antigen. *Br. med. J.*, iii, 605.

LO GERFO, P., KRUPEY, J. & HANSEN, H. J. (1971) Demonstration of an Antigen Common to Several Varieties of Neoplasia. *New Engl. J. Med.*, **285**, 138.

LO GERFO, P., LO GERFO, F., HERTER, F., BARKER, H. G. & HANSEN, H. J. (1972) Tumor-associated Antigen in Patients with Carcinoma of the Colon. *Am. J. Surg.*, **123**, 127.

MOERTEL, C. G. (1973) Large Bowel. *Cancer Medicine*, Chap. XXIV. Ed. J. F. Holland and E. Fry. Philadelphia: Lea and Febiger.

ROLLASON, J. G., PINCHERLE, G. & ROBINSON, D. (1972) Serum $\gamma$-glutamyl Transpeptidase in Relation to Alcohol Consumption. *Clin. chim. Acta*, **39**, 75.

ROSALKI, S. B. & RAU, D. (1972) Serum $\gamma$-glutamyl Transpeptidase in Alcoholism. *Clin. chim. Acta*, **39**, 41.

ZAMCHECK, N., MOORE, T. L., DHAR, P. & KUPCHIK, H. (1972) Immunological Diagnosis and Prognosis of Human Digestive-tract Cancer: Carcinoembryonic Antigens. *New Engl. J. Med.*, **286**, 83.